# Role of p120 Catenin in Epac1-Induced Chronic Postsurgical Pain in Rats

**DOI:** 10.1155/2019/9017931

**Published:** 2019-02-03

**Authors:** Peng Pan, Sai-Sai Huang, Shi-Ren Shen, Cui-E. Lu, Yi-Bin Qin, Jia-Long Zhang, Su Cao

**Affiliations:** ^1^Nantong University, No. 19 Qixiu Road, Nantong, Jiangsu 226001, China; ^2^Department of Anesthesiology, Affiliated Hospital of Nantong University, No. 20 Xisi Road, Nantong, Jiangsu 226001, China

## Abstract

Chronic postsurgical pain (CPSP) is a chronic pain state that is difficult to be treated clinically. A series of complicated changes have been produced from nociceptive stimulation to the occurrence and development of postsurgical pain. Many mechanisms remain unclear. In order to study the role of intercellular gap junctions in inducing inflammatory microenvironment at the beginning of nociceptor after operation, the model of skin/muscle incision and retraction (SMIR) was established. We observed the changes of the expression of exchange proteins directly activated by cAMP-1 (Epac1) and p120 catenin (p120), the quantities of macrophages and endothelial cells, vascular endothelial permeability, and mechanical withdrawal threshold (MWT). It was found that macrophages and endothelial cells were functionally coupled through Epac1-p120. Adhesive linkage disorder remodeled the chronic, inflammatory, and eutrophic microenvironment at the beginning of nociceptor after operation through macrophages, endothelial cells, and endothelial paracellular pathways. It might be an early event and a key step in peripheral sensitization of CPSP. The expression of p120 in muscle tissue around the incision might become a prognostic marker for the conversion of acute postsurgical pain into CPSP. Targeted intervention of Epac1-p120 might be a clinical strategy for inhibiting the conversion of acute postsurgical pain into CPSP.

## 1. Introduction

In the persistent postsurgical pain, there is a series of complicated changes from nociceptive stimulation to the occurrence and development of postsurgical pain. It is generally believed that peripheral sensitization and central sensitization caused by changes of neuronal plasticity are the mechanisms of chronic pain. Therefore, there is an urgent need to solve the target of peripheral sensitization for chronic postsurgical pain.

Gap junction (GJ), which is a special membrane network structure formed by connecting adjacent cells of the same type and different type, is a direct path to the exchange of material and information, the basic unit of which is connexin (Cx), playing a key role in cell movement from migration to metastatic invasion [1, 2] and regulating cell metabolism, internal environment stability, and other physiological processes. The main components of tight junctions are zonula occludens-1 (ZO-1) and claudin-5 [3, 4]. Vascular endothelial-cadherin (VE-cadherin) and p120 constitute the cadherin-catenin complex (CCC) [1, 5], which is dynamically regulated by many intracellular and extracellular signals [6]. Every kind of molecular change may affect CCC's stability; CCC is involved in regulating a series of biological behavior changes such as cell adhesion, migration, invasion, and proliferation. Cyclic adenosine monophosphate (cAMP) regulates intercellular adhesion, migration, and inflammation through Epac [7]. There are two subtypes of Epac: Epac1 and Epac2; Epac1 controls intercellular adhesion, links [8, 9], and regulates endothelial permeability barriers under injury [10]. This study previously found that Epac1 was expressed in macrophages and was not expressed in endothelial cells, while CPSP was associated with the extracellular signal-regulated kinases (ERKs) [11]. So what mechanism does Epac1 regulate the endothelial permeability barrier to be correlated with CPSP? Activation of muscle nociceptor can cause central sensitization more than that of skin nociceptor [12].

In this experiment, the SMIR model [13] was established to observe the changes of the expression of Epac1 and p120, the quantities of macrophages and endothelial cells, and vascular endothelial permeability around the incisional muscle and to study the role of intercellular gap junctions in conversion of Epac1-induced acute postsurgical pain into chronic pain.

## 2. Materials and Methods

### 2.1. Animals

Male Sprague-Dawley (SD) rats of weight 200–250 g and 8–10 weeks of age were provided by the Experimental Animal Center of Nantong University, approved by the Experimental Animal Protection and Care Committee of Nantong University. Three days before the experiment, the rats were allowed to drink water and feed freely in the Laboratory of Animal Behavior. The ratio of light to darkness was l2 h : 12 h, with the temperature at 23 ± 1°C and the humidity of 55–60%. The rats were fed with postoperative routine, which did not have any wound infection, hair removal, diarrhea, or other symptoms. A total of 170 rats were used in our present study.

### 2.2. SMIR Model and Injection of Drugs

Six groups were divided randomly. Naive group: no treatment was performed. Sham group: after anesthetized under isofluration induction with 3-4% induction and 1-2% maintenance and fixed in the supine position, the rats were cut at the right hind thigh, 3-4 mm interior of the middle medial saphenous vein. SMIR group: the cut was bluntly separated 7 to 10 mm by exposing the superficial muscles of the legs, with the tip of the retractor placed under the superficial muscle to expand the cut to 2 cm, which was sutured 1 h later. 8-pCPT group (Epac1 agonist group): the normal rats were injected at the right hind paw with Epac1 agonist 8-pCPT (ABNUS, China) of 1 *μ*g. SMIR + CE3F4 group (Epac1 inhibitor + SMIR Group): 7 days after SMIR modeling, Epac1 inhibitor CE3F4 of 1 *μ*g (Tocris, UK) was injected into the right hind paw. Solvent group: the right hind paw of rats was injected with the same dose of saline.

### 2.3. Behavioral Testing

According to the method of Dixon [14], the mechanical withdrawal threshold (MWT) was measured before surgery and on the 1st, 3rd, 7th, 14th, and 28th day after surgery. The rats were adapted for 30 min on the metal screen in the organic glass box (22 × 12 × 22 cm^3^). In the resting state, the Von Frey filament cilia stimulator (North Coast Medical, USA) with a stiffness of logarithmically shifted (0.16 g–26 g) was used to perform vertical stimulation to the hind paw. With Von Frey slightly bent as the total force standard, the duration was less than or equal to 4 s, and the appearance of paw withdrawal, paw licking, etc. was considered positive, otherwise negative. Starting from 0.16 g, the measurement was performed by a lifting method, and the results were recorded 5 times. If there were 2 or more reactions out of the 5 times, it was considered as a mechanical touch-induced pain. If there were no positive reactions at least 2 times, then the stimulation of the next large-degree force should be used. If there were 2 or more positive reactions, a smaller adjacent degree of stimulation strength should be given. This was continued until a positive or negative reaction straddle occurred, and measurements for 5 times were continuously taken with an interval of 30 s. The paw withdrawal threshold was measured against the threshold table and the unit was “g.” The lower the paw withdrawal threshold was, the more severe the mechanical hyperalgesia was.

### 2.4. Detection of Local Tissue Vascular Permeability

All rats were operated under isoflurane inhalation anesthesia with 3-4% induction and 1-2% maintenance. 2% Evans blue (Evan) solution of 1 ml/kg was slowly injected into the left femoral vein. 1 minute later, if the skin of the toes and ears turned blue, the injection was successful. After 60 minutes, the rats' chests were opened, and 100 to 200 ml of normal saline was perfused from the ascending aorta of the left ventricle, until a clear fluid flowed from auricula dextra. The same portion of the operated muscle tissue was taken out and placed in an EP tube for use. The 2% Evan was diluted 100-fold, and the concentration of 20 ng/ug of the liquid was serially diluted in half into 10, 5, 2.5, 1.25, 0.625, 0.313, and 0.156 ng/*μ*g, with formamide blank tubes in order to make an EB standard curve. The tissue was soaked in a 2% carboxamide bath at a rate of 100 mg : 1 ml, homogenized and placed in a 37°C incubator for 48 hours, and centrifuged at high speed (11,000 rpm) for 15 minutes, the supernatant of which was carefully aspirated and divided into three sample tubes (50 *μ*l/tube). Absorbance (A value) was measured at 620 nm in a microplate reader (BioTek USA). According to the standard curve, Evan content of the sample was calculated, and the mean value was recorded to detect the leakage outside the blood vessel. The unit was ng/mg.

### 2.5. Immunofluorescence Staining

All rats were anesthetized using isoflurane with 3-4% induction and 1-2% maintenance. Firstly, the blood was flushed with saline through the heart, and the tissue was fixed by perfusing 250 ml 4% paraformaldehyde. We collected the tissue and dehydrated them in 20% and 30% sucrose solution in turn. After the tissues were submerged, they were frozen and continuously sliced to a thickness of 6 *μ*m and stored at –20°C. The appropriate number of slices were randomly selected and washed in 0.01 mol/L PBS. The sections were blocked with 5% serum antibody blocking solution for 2 h at room temperature. Primary antibodies were diluted with 1% serum antibody dilution. Slices were incubated independently with Epac1 (1 : 50, Santa, USA), p120 (1 : 50, Santa, USA), CD34 (1 : 100, Abcam, UK), and CD68 (1 : 50, Abcam, UK). After incubating at 4°C overnight, sections were washed with 0.01 mol/L PBS for 10 min × 3 times. Secondary antibodies were diluted with 0.01 mol/L PBS : Cy3-goat anti-rabbit IgG (1 : 1000, Jackson, USA) and FITC-goat anti-mouse IgG (1 : 1000, Jackson, USA). The tissue sections were incubated with the corresponding secondary antibody and incubated in the dark for 2 hours at room temperature. After rinsing for 15 min × 3 times with 0.01 mol/L PBS, the sections were mounted under dark conditions. The films were photographed under a fluorescence microscope (OLYMPUS. Japan).

### 2.6. Local Tissue HE Staining

For HE staining, the tissue was collected for immunofluorescence staining, and the slices were incubated in a 37°C incubator, which were soaked in water for about 30–60 seconds. Firstly, the tissue was wetted with distilled water for 1-2 minutes before staining to ensure that the whole tissue was moistened and evenly distributed. Secondly, the tissue was stained with a nuclear stain for about 5 minutes, washed with water for 3–5 seconds, rinsed with 2 drops in dichroic agent, and then flushed with water. Thirdly, the tissue was stained with a paddle stain for 2 minutes, dehydrated according to a gradient (30% ethanol, 70% ethanol, 95% ethanol, and anhydrous ethanol), blotted dry, and mounted microscopically. Lastly, light microscopy was used to observe local tissue capillary dilation, rupture, and erythrocyte detachment.

### 2.7. Western Blotting Analyses

Rats were anesthetized as previously described, and L3-L5 spinal lumbar enlargement was harvested. Whole cell lysis assay (KeyGEN, China) was used for the tissue samples extraction, and the mixture was homogenized using the Eppendorf 5417R Refrigerated Microcentrifuge (Eppendorf, Hamburg, Germany). Cell lysates were cleared by centrifugation at 4.0°C and 12,000 rpm and stored at 4.0°C until analyzed using sodium dodecyl sulfate-polyacrylamide gel electrophoresis. For separation, 30 *μ*g total protein per gel lane was loaded onto 10% gels (Beyotime Institute of Technology, Shanghai, China). The separated proteins were then transferred onto 0.45 mm polyvinylidene fluoride membranes using an electrophoresis and transfer system (Bio-Rad Laboratories, Hercules, CA, USA). Membranes were incubated for 2.0 h at room temperature in tris-buffered saline and Tween-20 blocking solution containing 5.0% skim milk powder, followed by overnight incubation at 4.0°C in blocking solution containing primary antibodies against the following proteins: Epac1 (1 : 300, Santa, USA), p120 (1 : 300, Santa, USA), and *β*-actin (1 : 5000, Sigma, USA). Membranes were washed three times with Tris-buffered saline and Tween 20 (10 min/wash) and incubated at room temperature for 2.0 h with the appropriate secondary antibody (1 : 5000, Jackson, USA). After washing, immunolabeling was detected using the Tanon 2500 gel imaging system (Yph-Bio Co Ltd, Beijing, China) and hypersensitive ECL chemiluminescence detection kit (Absin, China). The results were analyzed using Image J (National Institutes of Health, Bethesda, MD, USA) with *β*-actin as an internal reference. Relative expression was calculated as the gray value ratio of the target protein to *β*-actin.

### 2.8. Data and Statistical Analyses

Experimental data were analyzed using SPSS 20.0 software. Measured data were expressed as mean ± SEM, and the behavioral data were analyzed by 2-way analysis of variance (ANOVA) followed by the Bonferroni test as the multiple comparison analysis. Image J was used to analyze fluorescence intensity, and one-way analysis of variance was used for comparison between groups. A value of *p* < 0.05 was considered statistically significant.

## 3. Results

### 3.1. Establishment of SMIR Model in Rats and Detection of MWT

**Figure 1:**
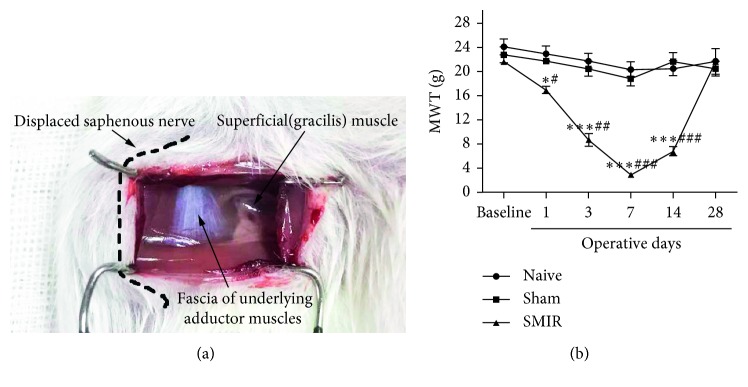
Establishment of the SMIR model in rats and detection of MWT. (a) SMIR model was established, and the dashed line represents the saphenous nerve that had been pulled and translocated. Adductor fascia was seen white in the cut. (b) MWT of the SMIR group was significantly reduced time dependently on the 1st, 3rd, 7th, and 14th day after operation; however, the 14th day after operation began to grow up. ^*∗*^*P* < 0.05, ^*∗∗∗*^*P* < 0.001 vs. naive group; #*P* < 0.05, ##*P* < 0.01, ###*P* < 0.001 vs. sham group. Data information in (b): error bars indicate SEM derived from at least three independent experiments, and significance was calculated by 2-way analysis of variance (ANOVA) followed by the Bonferroni test (*n*=5).

In order to study the peripheral mechanism of postoperative chronic pain, a model was established according to the literature. After stimulation of the right hind paw of rats with the Von Frey filament cilia stimulator, the MWT of the SMIR group decreased significantly in a time-dependent manner. The MWT was significantly decreased on the 7th day, increased on the 14th day, and returned to normal on the 28th day after operation. It was found that SMIR after operation showed persistent hyperalgesia (allodynia), and the SMIR model was successfully prepared (Figure 1).

### 3.2. CPSP Correlated with the Continuous Increase of the Vascular Endothelial Permeability and the Distribution of Macrophages and Endothelial Cells around the Incision

**Figure 2:**
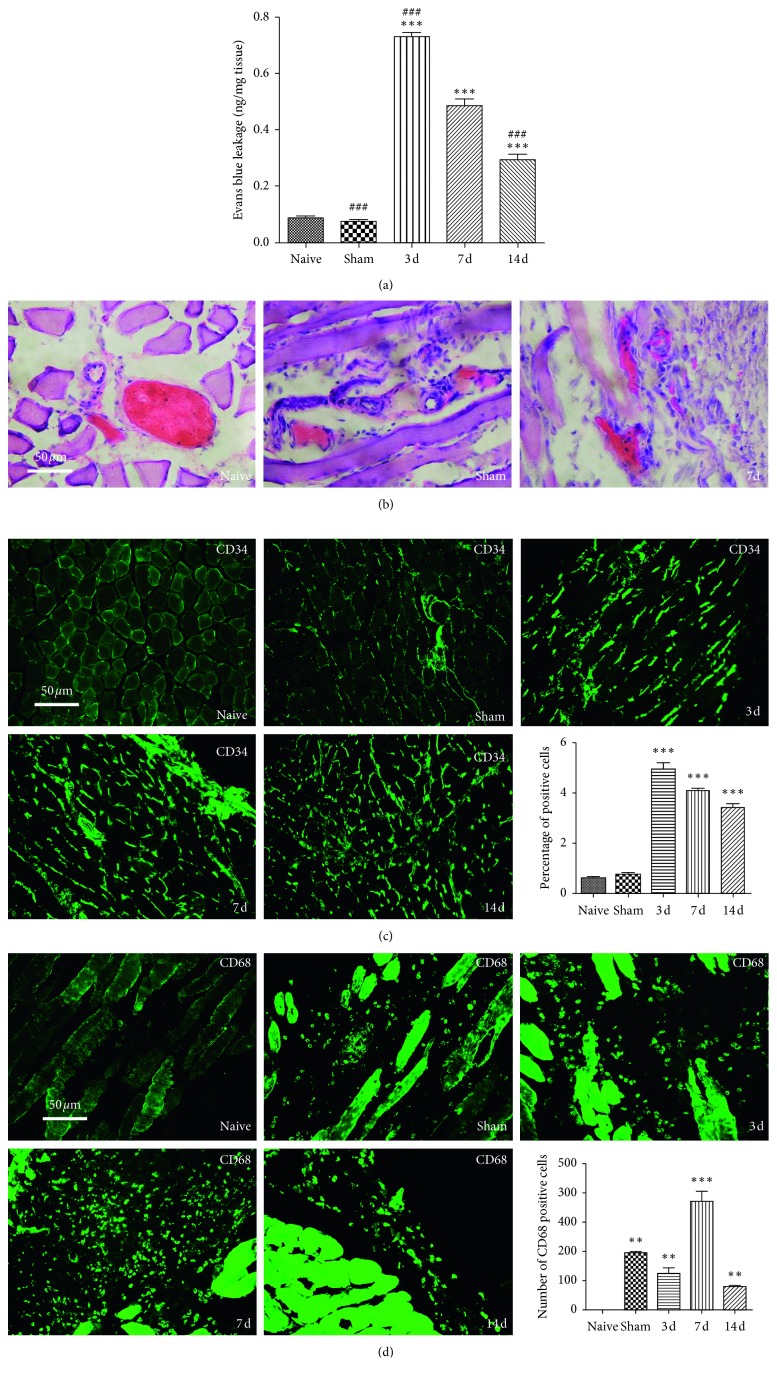
CPSP was correlated with the continuous increase of the vascular endothelial permeability and the distribution of macrophages and endothelial cells around the incision. (a) The intravascular Evan extravasation of the SMIR group was significantly increased after surgery, ^*∗∗∗*^*P* < 0.001 vs. naive group. In the SMIR group, the intravascular Evan extravasation on the 3rd day increased significantly, while it decreased significantly on the 14th day; in the Sham group also, it decreased (###*P* < 0.001 vs. the 7th day). (b) The wall of the fine and small blood vessels in the SMIR and sham groups was ruptured and incomplete, the distribution of red blood cells in the intravascular density narrowed, and the distribution of red cells increased in the interstitial space. (c) Endothelial cells increased significantly on the 3rd, 7th, and 14th day in the SMIR group, ^*∗∗∗*^*P* < 0.001 vs. naive group. (d) Macrophages increased significantly in the sham group and the 3rd, 7th, and 14th day in the SMIR group, ^*∗∗*^*P* < 0.01, ^*∗∗∗*^*P* < 0.001 vs. naive group. Data information in (a, c, d): error bars indicate SEM derived from at least three independent experiments, and significance was calculated by one-way analysis of variance (*n*=5).

In this experiment, in order to study the role of macrophages and endothelial cells in the remodeling of inflammatory microenvironment, the muscle tissue around the incision was taken on the 3rd, 7th, and 14th day after SMIR to detect the vascular endothelial permeability and the distribution of macrophages and endothelial cells. It was found that surgical wounds and repairs induced obstruction of the vascular endothelial barrier, maintenance of endothelial hyperpermeability, and increased distribution of macrophages and endothelial cells in muscle tissue around the incision in rats, and macrophages were not distributed in normal rats (Figure 2).

### 3.3. Effects of CPSP on Expression of Epac1 and p120 in Muscle Tissue around Postoperative Incision and Lumbar Spinal Cord

**Figure 3:**
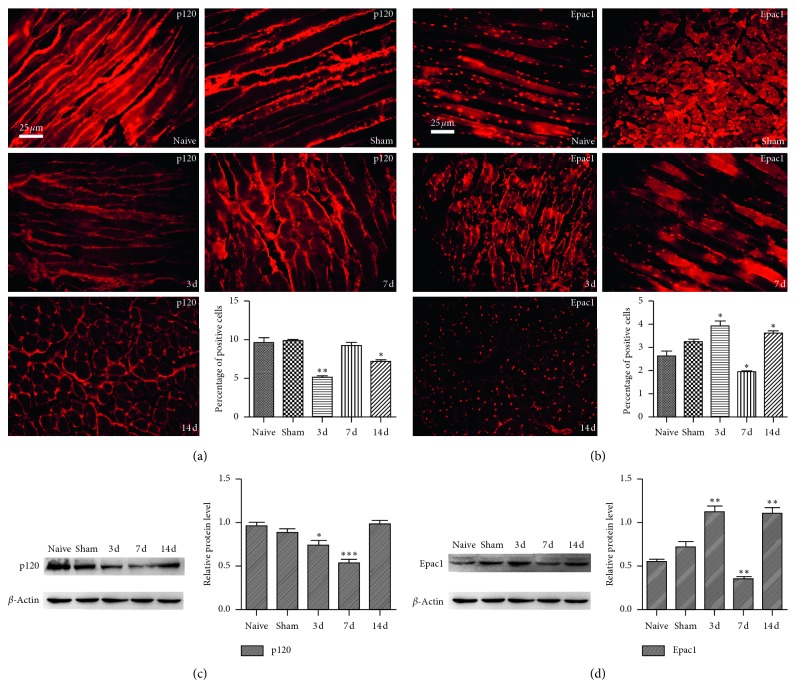
Effects of CPSP on expression of p120 and Epac1 in muscle tissue around postoperative incision and lumbar spinal cord. (a) The expression of p120 in muscle tissue was decreased significantly on the 3rd and 14th day in the SMIR group, ^*∗*^*P* < 0.05, ^*∗∗*^*P* < 0.001 vs. naive group. (b) The expression of Epac1 in muscle tissue was increased dramatically on the 3rd and 14th day and was decreased significantly on the 7th day after operation in the SMIR group, ^*∗*^*P* < 0.05 vs. naive group. (c) The expression of p120 in the spinal cord was decreased significantly on the 3rd and 7th day in the SMIR group, ^*∗*^*P* < 0.05, ^*∗∗∗*^*P* < 0.001 vs. naive group. (d) The expression of Epac1 in spinal cord was increased dramatically on the 3rd and 14th day and was decreased significantly on the 7th day after operation in the SMIR group, ^*∗∗*^*P* < 0.01 vs. naive group. Data information in (a-b): error bars indicate SEM derived from at least three independent experiments, and significance was calculated by one-way analysis of variance (*n*=5).

In order to elucidate the role and regulatory mechanism of GJ in peripheral sensitization of CPSP, the changes of Epac1 and p120 expression in peripheral muscle tissues around the incision and lumbar spinal cord were detected on the 3rd, 7th, and 14th day after SMIR, which was compared with naive. It was found that surgical wounds and repairs induced the low expression of p120. Epac1 was highly expressed, showing dynamic changes in activation and reactivation(Figure 3).

### 3.4. Immunofluorescence Staining Presenting the Locations of Epac1 and p120 in Macrophages and Endothelial Cells

**Figure 4:**
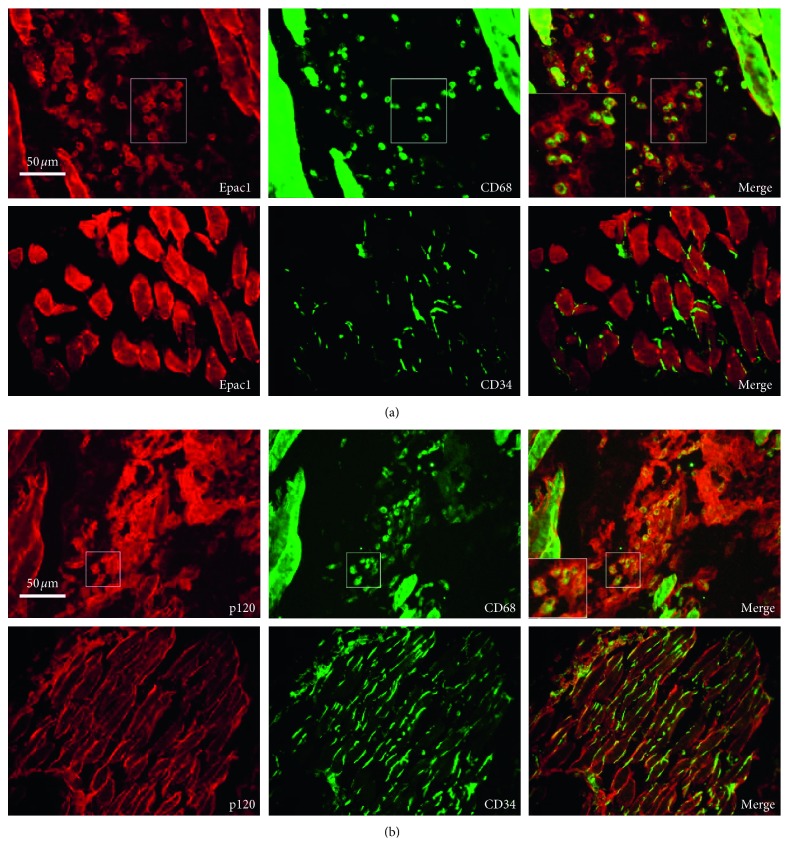
Immunofluorescence staining presented the locations of Epac1 and p120 in macrophages and endothelial cells. (a) Epac1 was colabeled with macrophage marker CD68 and did not colabel with the endothelial cell marker CD34. (b) p120 was colabeled with the macrophage marker CD68 and the endothelial cell marker CD34.

In order to study the distribution of Epac1 and p120, target cells of Epac1 and p120 expression were detected. It was found that p120 was distributed in macrophages and endothelial cells. Epac1 was distributed in macrophages, and there was no distribution of Epac1 in endothelial cells (Figure 4).

### 3.5. Effects of 8-pCPT on Changes of MWT, Vascular Endothelial Permeability, Macrophages, Endothelial Cells, and the Expression of p120 in Muscle Tissue around the Incision

**Figure 5:**
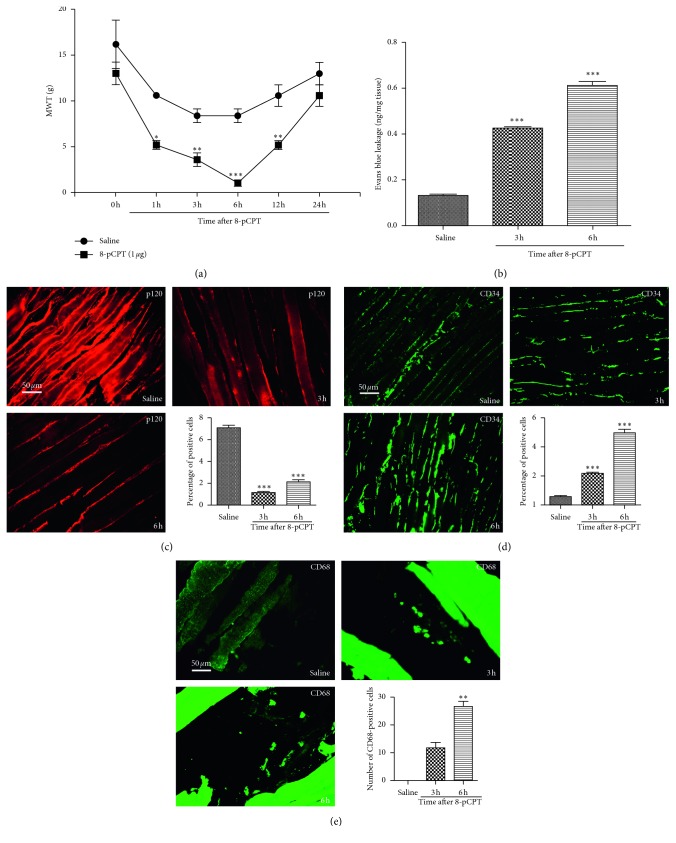
Effects of 8-pCPT on changes of MWT, vascular endothelial permeability, macrophages, endothelial cells, and the expression of p120 in muscle tissue around the incision. (a) MWT at the 1st, 3rd, 6th, and 12th hour in the 8-pCPT group was significantly lower, ^*∗*^*P* < 0.05, ^*∗∗*^*P* < 0.01, ^*∗∗∗*^*P* < 0.001 vs. saline group. (b) The vascular endothelial permeability of the 8-pCPT group at the 3rd and 6th hour was significantly increased, ^*∗∗∗*^*P* < 0.001 vs. saline group. (c) p120 was significantly decreased in the 8-pCPT group at the 3rd and 6th hour, ^*∗∗∗*^*P* < 0.001 vs. saline group. (d) The endothelial cells in the 8-pCPT group significantly increased at the 3rd and 6th hour, ^*∗∗∗*^*P* < 0.001 vs. saline group. (e) Macrophages in the 8-pCPT group significantly increased at the 3rd and 6th hour, ^*∗∗*^*P* < 0.01 vs. saline group. Data information (a–e) error bars indicate SEM derived from at least three independent experiments, (a) significance was calculated by 2-way analysis of variance (ANOVA) followed by Bonferroni test, and (b–e) significance was calculated by one-way analysis of variance (*n*=5).

In order to elucidate the mechanism of Epac1 in promoting the conversion of acute postsurgical pain into CPSP, Epac1 agonist 8-pCPT was injected into the right hind paw of normal rats to observe the changes of MWT and vascular endothelial permeability, macrophages, endothelial cells, and the expression of p120 around the incision of muscle tissue. It was found that activation of Epac1 was related to hyperalgesia, high permeability of vascular endothelium, low expression of p120, and increased distribution of macrophages and endothelial cells (Figure 5).

### 3.6. Effects of CE3F4 on Changes of MWT, Vascular Endothelial Permeability, Macrophages, Endothelial Cells, and the Expression of p120 by CPSP in Muscle Tissue around the Incision

**Figure 6:**
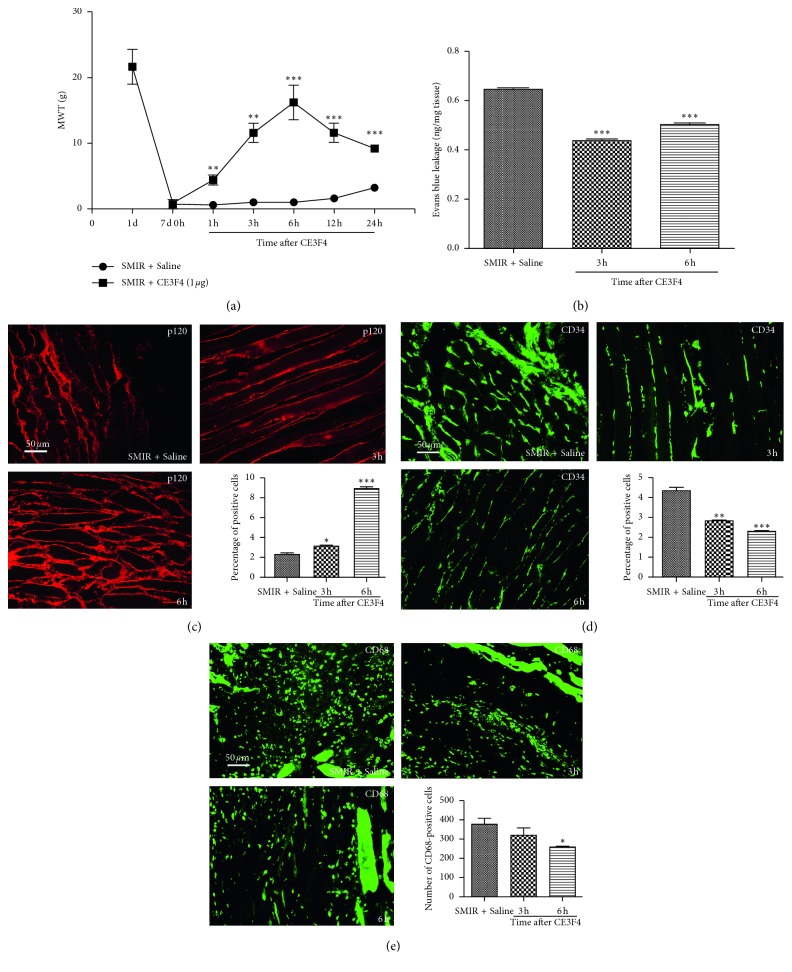
Effects of CE3F4 on changes of MWT, vascular endothelial permeability, macrophages, endothelial cells, the expression of p120 by CPSP in muscle tissue around the incision. (a) MWT at the 1st, 3rd, 6th, 12th and 24th hour in SMIR + CE3F4 group was significantly higher, ^*∗∗*^*P* < 0.01, ^*∗∗∗*^*P* < 0.001 vs. SMIR + saline group. (b) Evan's exudation was significantly reduced at the 3rd and 6th hour in SMIR + CE3F4 group, ^*∗∗∗*^*P* < 0.001 vs. SMIR + saline group. (c) The expression of p120 at the 3rd and 6th hour in SMIR + CE3F4 group was significantly increased, ^*∗*^*P* < 0.05, ^*∗∗∗*^*P* < 0.001 vs. SMIR + saline group. (d) Endothelial cells were significantly decreased at the 3rd and 6th hour in SMIR + CE3F4 group, ^*∗∗*^*P* < 0.01, ^*∗∗∗*^*P* < 0.001 vs. SMIR + saline group. (e) Macrophages were significantly reduced at 6th hour in SMIR + CE3F4 group, ^*∗*^*P* < 0.05 vs. SMIR + saline group. Data information: (a–e) error bars indicate SEM derived from at least three independent experiments, (a) significance was calculated by 2-way analysis of variance (ANOVA) followed by the Bonferroni test, and (b–e) significance was calculated by one-way analysis of variance (*n*=5).

In order to elucidate the mechanism of Epac1 in promoting the conversion of acute postsurgical pain into CPSP, the Epac1 inhibitor CE3F4 was injected on the 7th day after SMIR to observe the changes of MWT, vascular endothelial permeability, macrophages, endothelial cells, and the expression of p120 around the incision of muscle tissue. It was found that inhibition of Epac1 was related to the reduction of hyperalgesia, decreased endothelial permeability, high expression of p120, and decreased distribution of macrophages and endothelial cells (Figure 6).

## 4. Discussion

SMIR stretched the skin and muscle tissue for a long time, without damaging the main peripheral nerve around the incision (Figure 1(a)), which only induced sustained postoperative mechanical touch-induced pain to reflect the characteristics of the inflammatory microenvironment of the surgical field and postcontinuous pain process [13]. In this experiment, the time dependence of MWT after SMIR was significantly decreased (Figure 1(b)), showing that the SMIR rat model was successfully prepared.

Sustained hyperglycemia contributed to the increase of endothelial cells and macrophages [15]. The macrophages which were recruited to the site of injury released a variety of proinflammatory cytokines such as interleukin-1*β* (IL-1*β*) and tumor necrosis factor *α* (TNF-*α*) [16], which had the functions of removing cell debris and apoptotic bodies [1], phagocytizing necrotic substances, releasing CCL2, CX3CL1, and other chemokines [17], and promoting muscle healing [18] and immune cell infiltration [19]. Stimulation of macrophages could induce endothelial cell inflammatory response [20].

Endothelial cells regulated leukocyte activity, inflammation, and platelet aggregation by secreting various active substances [10], while endothelial hyperpermeability induced inflammatory response in local tissues [21]. In this experiment, it was found that the exudation of Evans blue continued to increase in the muscle around the incision of the SMIR group (Figure 2(a)), and the thin and small vascular walls were incomplete (Figure 2(b)). The distribution of red blood cells (Figure 2(b)), endothelial cells (Figure 2(c)), and macrophages (Figure 2(d)) continued to increase in the interstitial space. It was suggested that surgical injury and repairment continued to promote the differentiation of monocytes into mature macrophages, which were recruited to the site of surgical injury, promoting endothelial cell proliferation, endothelial barrier dysfunction, and high permeability of vascular endothelium. It was similar to the mechanism of intravascular Evans blue and erythrocyte exudation, and the infiltration and distribution of intravascular leucocytes, glucose, albumin, and inflammatory mediators might continue to increase in the intramuscular space around the incisional incision. A vicious circle might be developed between proliferation of endothelial cells and recruitment of proinflammatory cytokines and chemokines released by macrophages at the site of injury, remodeling local chronic, inflammatory, nutrient-rich microenvironment at the beginning of postoperative nociceptors.

The decrease of cell adhesion was associated with cell movement [2]. p120 mediated cytoskeletal rearrangement and changed gap distance between cells [22], which played a key role in remodeling tissue function and cell morphology [23, 24]. In this experiment, the expression of p120 was found to be significantly reduced in the SMIR group (Figure 3). p120 was localized to endothelial cells and macrophages (Figure 4(b)). Surgical injury and repairment contributed to the instability of adhesion links, which might play a key role in the remodeling of local tissue, cell morphology and function by regulating the characteristics of the cell membrane, the exchange of intercellular material, and the invasion of cells.

Epac1 was a proinflammatory factor [25], which was associated with acute inflammatory pain turning into chronic inflammatory pain through ERK [26], and it could be used as a target for the treatment of chronic pain [10]. The low expression of p120 contributed to high permeability of vascular endothelium [27, 28], and endothelial hyperpermeability induced pain [29]. In this experiment, the expression of Epac1 increased significantly (Figure 3) in the SMIR group, which was localized to macrophages without localization of endothelial cells (Figure 4(a)). Activation of Epac1 with 8-pCPT was associated with mechanical allodynia in rats (Figure 5(a)), high vascular endothelial permeability (Figure 5(b)), and low expression of p120 (Figure 5(c)), and endothelial cells (Figure 5(d)) and macrophages (Figure 5(e)) were significantly increased. Inhibition of Epac1 with CE3F4 reversed CPSP-induced mechanical hyperalgesia in rats (Figure 6(a)), decreased endothelial permeability (Figure 6(b)), increased the expression of p120 (Figure 6(c)), endothelial cells (Figure 6(d)), and macrophages (Figure 6(e)) were significantly reduced. It was suggested that CPSP was associated with Epac1-p120 activated by surgical injury and repairment. Adhesive link disorder was the key step to maintain the high permeability of vascular endothelial cells after operation. Tight links were not involved in pathological changes of vascular endothelial permeability after surgery. Evaluating the expression of p120 in muscle tissue around the incision might become a prognostic marker for the conversion of acute postsurgical pain into CPSP.

Poorly repaired wounds induced chronic inflammatory reactions at the site of surgical injury, resulting in the occurrence of CPSP [30]. In the process of postoperative injury and repairment, the maintenance of high permeability of vascular endothelial cells in peripheral muscle tissue, the continuous increase of macrophages and endothelial cells infiltration, and adhesion link disorder were the necessary steps to induce the repairment of wound and functional reconstruction after operation. The complex pathophysiology of chronic mild hypoxia could play an important role in promoting peripheral sensitization of CPSP.

Inducing inflammatory response of macrophages to produce abnormal inflammatory pain [31–33] and inducing anti-inflammatory cytokines to produce analgesia [18] might be a useful target for the development of novel therapeutics [34]. In this experiment, it was found that the infiltration and distribution of macrophages in the muscle around incisions were increased (Figure 2(d)), but there was no significant change in vascular endothelial permeability (Figure 2(a)) and the expression of p120 and Epac1 (Figure 3) in the sham group. It was suggested that the increase of infiltration and distribution of macrophages could not induce the local inflammatory microenvironment with neuronal plasticity. Epac1 and p120 might be useful targets for clinical development of therapeutics.

P120 regulated adhesion and tight links [35], and adhesion linkage disorder led to high permeability of vascular endothelium through the paracellular pathway [36]. In the early stage of acute lung injury (ALI), the increase of transmembrane permeability was earlier than that of paracellular permeability [37]. Intervention of transcellular transport might be an effective strategy to prevent pulmonary edema in ALI [38–40]. In this experiment, p120 was localized in endothelial cells (Figure 4(b)) and was colocalized with Epac1 in macrophages. It was suggested that surgical injury and repairment activated Epac1-p120. Dissociated adhesion links through low expression of p120 induced metastatic invasion of macrophage and endothelial barrier dysfunction. Macrophages and endothelial cells were functionally coupled via p120, and the translocation of macrophages, endothelial cells, and paracellular endothelial cells induced the high permeability of vascular endothelial cells, remolding local chronic, inflammatory, and eutrophic microenvironment after operation. They were one of the initiating cells and effector cells of peripheral sensitization of CPSP. Adhesion link dysfunction and low expression of p120 might be the key link and initial event of peripheral sensitization of CPSP. Regulation of transcellular transport through targeted intervention of p120 might be a strategy to inhibit peripheral sensitization and provided new ideas for the treatment of CPSP.

## 5. Conclusion

Macrophages and endothelial cells were functionally coupled through Epac1-p120. Epac1 and p120 were coexpressed with macrophages. Adhesive linkage disorder remodeled the chronic, inflammatory, and eutrophic microenvironment at the beginning of nociceptor after operation through macrophages, endothelial cells, and endothelial paracellular pathways. It might be an early event and a key step in peripheral sensitization of CPSP. Evaluating the expression of p120 in muscle tissue around the incision might become a prognostic marker for the conversion of acute postsurgical pain into CPSP. Targeted intervention of Epac1-p120 might be a clinical strategy for inhibiting the conversion of acute postsurgical pain into CPSP.

## Data Availability

The data used to support the findings of this study are available from the corresponding author upon request.
